# Honokiol Improves Liver Steatosis in Ovariectomized Mice

**DOI:** 10.3390/molecules23010194

**Published:** 2018-01-17

**Authors:** Yeon-Hui Jeong, Haeng Jeon Hur, Eun-Joo Jeon, Su-Jin Park, Jin Taek Hwang, Ae Sin Lee, Kyong Won Lee, Mi Jeong Sung

**Affiliations:** 1 Division of Nutrition and Diet, Korea Food Research Institute, Jeollabuk-Do 55365, Korea; Jeong.Yeon-hui@kfri.re.kr (Y.-H.J.); mistltoe@kfri.re.kr (H.J.H.); Jeon.Eun-ju@kfri.re.kr (E.-J.J.); Park.Su-jin@kfri.re.kr (S.-J.P.); jthwang@kfri.re.kr (J.T.H.); 2 Division of Functional Food Research, Korea Food Research Institute, Jeollabuk-Do 55365, Korea; aslee@kfri.re.kr (A.S.L.); lkw@kfri.re.kr (K.W.L.)

**Keywords:** honokiol, nonalcoholic fatty liver disease, ovariectomy, postmenopausal, liver steatosis

## Abstract

Nonalcoholic fatty liver disease (NAFLD) is the most common liver disease, and is associated with the development of metabolic syndrome. Postmenopausal women with estrogen deficiency are at a higher risk of progression to NAFLD. Estrogen has a protective effect against the progression of the disease. Currently, there are no safe and effective treatments for these liver diseases in postmenopausal women. Honokiol (Ho), a bioactive natural product derived from Magnolia spp, has anti-inflammatory, anti-angiogenic, and anti-oxidative properties. In our study, we investigated the beneficial effects of Ho on NAFLD in ovariectomized (OVX) mice. We divided the mice into four groups, as follows: SHAM, OVX, OVX+β-estradiol (0.4 mg/kg of bodyweight), and OVX+Ho (50 mg/kg of diet). Mice were fed diets with/without Ho for 12 weeks. The bodyweight, epidermal fat, and weights of liver tissue were lower in the OVX group than in the other groups. Ho improved hepatic steatosis and reduced proinflammatory cytokine levels. Moreover, Ho markedly downregulated plasma lipid levels. Our results indicate that Ho ameliorated OVX-induced fatty liver and inflammation, as well as associated lipid metabolism. These findings suggest that Ho may be hepatoprotective against NAFLD in postmenopausal women.

## 1. Introduction

Nonalcoholic fatty liver disease (NAFLD), ranging from benign steatosis to nonalcoholic steatohepatitis (NASH), is the most common cause of chronic liver disease. It is characterized by abnormal fat accumulation in the hepatocytes [1]. NAFLD has a high incidence worldwide. The prevalence of NAFLD in Western countries ranges from 20% to 40% in adults and between 10% and 20% in Asian countries [2]. NAFLD is supposed to be a hepatic symptom of metabolic disorder, and is associated with metabolic disease characteristics, such as insulin resistance, dyslipidemia, and obesity [3,4].

Postmenopausal women with reduced circulating estrogen levels develop increased body weights, hyperlipidemia, insulin resistance, and visceral fat accumulation [5,6]. Menopause may induce and accelerate the development of NAFLD and obesity [7,8]. Some studies have reported that estrogen has a hepatoprotective effect against the progression of NAFLD in females. Furthermore, women can spend a substantial portion of their lives in the postmenopausal state because of the dramatic increase in lifespan. Therefore, dietary therapy to decrease body weight may help prevent the acceleration of NAFLD. However, there are no specific diets or drugs for the treatment of this liver disorder [9].

Honokiol (Ho) is a bioactive compound obtained from Magnolia officinalis that has been used without remarkable toxicity as part of herbal medicines in Asian countries [10]. This compound possesses several potent pharmacological functions, including anti-tumor, anti-oxidative, and anti-inflammatory functions [11,12,13,14]. Furthermore, in vitro studies suggest that Ho attenuates lipid accumulation in the hepatocytes [15]. In addition, Zhong et al. reported the protective effects of Ho on abnormal hepatic lipid accumulation in mice subjected to high cholesterol and high-fat diets [16]. However, the beneficial effect of Ho on the pathogenesis of hepatic lipid accumulation in postmenopausal obesity is unknown.

Considering these observations, estrogen deficiency may exacerbate the intensity of NAFLD in postmenopausal women. However, the mechanisms underlying this progression have not yet been demonstrated in the development of NAFLD in ovariectomized (OVX) mice. Therefore, we investigated the effects of Ho in liver steatosis, and assessed its protective effect on NAFLD in post-menopausal women.

## 2. Results

### 2.1. Ho Reduces Bodyweight, Epidermal Fat, and Liver Weight in OVX Mice

**Figure 1:**
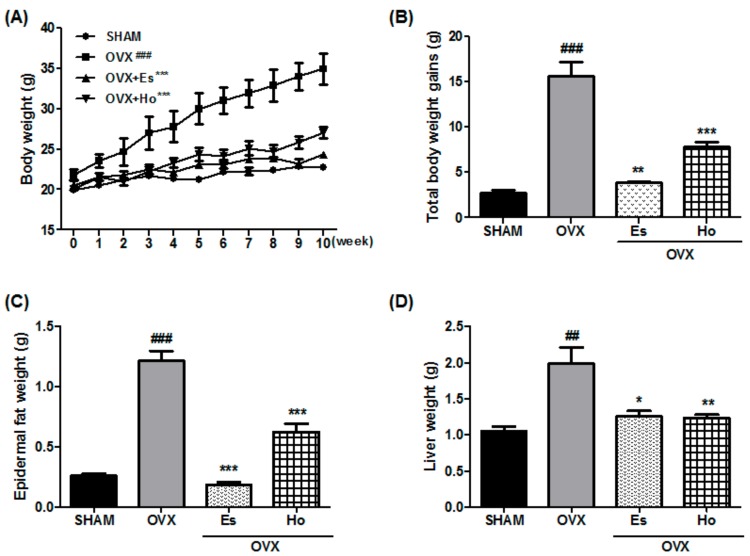
Honokiol reduces bodyweight, epidermal fat and liver weight. Data were determined after 11 weeks of administration with Honokiol, estradiol in female OVX mice. Body weight (**A**) and total body weight gain (**B**) change at 2 weeks after operation. Epidermal fat (**C**) weight and liver weight (**D**) changed after the administration of Honokiol and estradiol after OVX. Data shown are means ± standard error of mean (SEM). * *p* < 0.05, ** *p* < 0.01 and *** *p* < 0.001 vs. OVX-operated group. ^##^ *p* < 0.01 and ^###^ *p* < 0.001 vs. sham-operated group. OVX, ovariectomy; Es, estradiol.

After 12 weeks being fed a Ho diet, the OVX mice had markedly increased body weights and more body weight gain compared with SHAM mice. However, the body weights and body weight gain for Ho mice were markedly lower than those for OVX mice (Figure 1A,B). As shown in Figure 1C,D, Ho mice had substantially reduced epidermal fat and liver weights compared with OVX mice. Moreover, Es mice also had markedly decreased body weights, body weight gain, epidermal fat, and liver fat.

### 2.2. Ho Regulates Lipid Metabolism and Reduces Liver Injury Markers in OVX Mice

The accumulation of lipids and fat largely originates from circulating triglycerides and cholesterol. Total cholesterol (TC) and low-density lipoprotein-cholesterol (LDL-C) levels were markedly increased in the OVX group than in the SHAM group. Triglyceride (TG) levels increased and high-density lipoprotein-cholesterol (HDL-C) decreased in the OVX group compared with those in the SHAM group. In contrast, The OVX+Ho group had significantly lower TC, LDL-C, and TG levels compared to the OVX group. Although the OVX group did not experience a remarkable decrease in HDL-C, the OVX+Ho group experienced significantly decreased levels compared to those in the OVX group. The Es group exhibited levels similar to the those of the SHAM group (Table 1).

### 2.3. Ho Inhibits Hepatic Steatosis in OVX Mice

**Figure 2:**
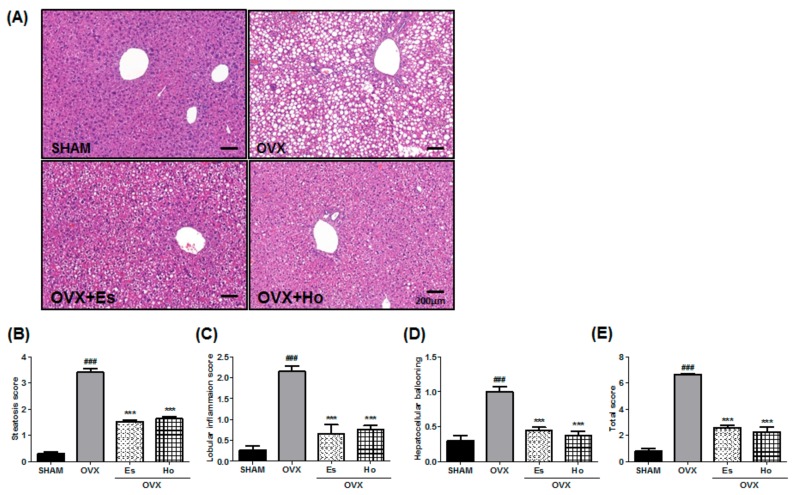
Honokiol intake decreased fat accumulation in the liver and hepatic steatosis. The livers were fixed with 4% formaldehyde and then embedded in paraffin. Sections were stained with hematoxylin and eosin, and examined by light microscope (**A**). (**B**) Steatosis score, (**C**) Lobular inflammation score, (**D**) Hepatocellular ballooning, and (**E**) total score were determined. Scale bar = 200 μm. Values are expressed as means ± SEM. ^###^ *p* < 0.001 vs. control group. *** *p* < 0.001 vs. OVX group.

We examined the liver phenotypes of the mice to estimate the effect of Ho on hepatic steatosis. In the OVX group, marked macrovascular steatosis, increases in the number of infiltrating inflammatory cells, and hepatocytes ballooning in the liver were observed. The OVX+Ho group exhibited reduced hepatic steatosis, inflammatory infiltration, and lobular ballooning (Figure 2A). Histological scoring of liver slides revealed that OVX+Ho dramatically ameliorated the hepatic steatosis scores (Figure 2B–E).

### 2.4. Ho Inhibits Hepatic Inflammatory Gene Expression in OVX Mice

**Figure 3:**
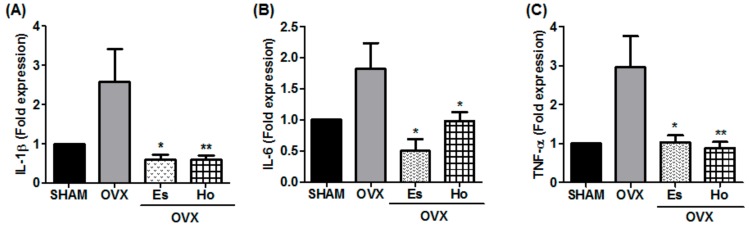
Honokiol inhibited liver inflammatory molecules in OVX mice. mRNA expressions in liver tissues from mice of each treatment groups were assessed using real time PCR analysis. Related expression of (**A**) IL-1β, (**B**) IL-6 and (**C**) TNF-α were normalized with the β-actin gene. * *p* < 0.05 and ** *p* < 0.01 vs. OVX group.

We performed an evaluation on the expression of inflammatory cytokines, such as TNF-α, IL-6, and IL-1β, to investigate the effect of Ho on the gene expression of hepatic inflammatory markers. Inflammatory genes for TNF-α, IL-6 and IL-1β were dramatically elevated in OVX mice compared with SHAM mice. However, those levels were significantly lower in OVX+Ho mice compared to OVX mice (Figure 3).

## 3. Discussion

NAFLD is the most common liver disorder, and is a serious public health concern. Postmenopausal women have more risk factors for progressing to NAFLD, because of a reduction in estrogen compared to premenopausal women [17,18,19]. Extensive evidence demonstrates that the loss of estrogen increases liver steatosis in humans with estrogen receptor α (ERα) mutations. In addition, the metabolic effects of estrogen in relation to body weight regulation and lipid accumulation are associated with ERα. Therefore, OVX mice lacking aromatase, which are removed by estrogen, develop hepatic steatosis. In addition, estrogen treatment improves hepatic steatosis in mice and humans [20,21,22]. Studies have reported that estrogen has a hepatoprotective effect against the progression of NAFLD in both humans and in mice. Furthermore, OVX mice are commonly used in research involving postmenopausal women. Many rodent studies have shown that OVX increases obesity and causes other metabolic syndromes, such as dyslipidemia and NAFLD [3,4]. The liver plays an important role in metabolism. In the presence of estrogen deficiency, there are changes in the regulation of proteins related to hepatic steatosis. Identifying and characterizing the proteins and molecular signals can potentially lead to the determination of molecular biomarkers for NAFLD in postmenopausal women [23]. In future studies, we should confirm the effect of Ho on changes in proteins to identify biomarkers of hepatic steatosis in OVX mice. In this study, OVX mice had increased body weight and developed liver steatosis. However, the administration of Ho reduced body weight and hepatic steatosis. Therefore, these results suggest that Ho ameliorates NAFLD in OVX mice.

Most patients with NAFLD have one or more risk factors, including hypertension, insulin resistance, obesity, and abnormalities in lipid metabolism, including elevated LDL-C and reduced HDL-C, which may be factors related to NASH development [24]. Circulating serum TG and FFA are stored in adipose tissue and liver [25,26,27]. The current findings showed that OVX mice experienced a marked increase in TG and LDL-C and a reduction in HDL-C levels. However, Ho treatment reversed those levels. In addition, histopathological changes were found during the examination of liver function. Aside from this, fat accumulation, ballooning degeneration, and infiltrating inflammatory cells were observed in the OVX group. Ho treatment attenuated these histopathological changes. Thus, the current study results suggest that the inhibition of lipid and liver injury may be associated with the protective effect of Ho on NAFLD in OVX mice.

Although the pathogenesis of NAFLD is not completely understood, a ‘two-hit’ hypothesis may help explain it. The ‘first hit’ is related to hepatic steatosis. The ‘second hit’ activates oxidative stress in addition to the production of inflammatory cascades, eventually leading to liver fibrosis [28,29]. Recently, however, this theory has become a ‘multi-hit’ theory, including insulin resistance, obesity with adipocyte production, intestinal microbiota, dietary and genetic factors. All these factors lead to hepatic inflammation [30]. The process of NAFLD development closely correlates with postmenopausal and circulating proinflammatory mediators, which are TNF-α, IL-1β, and IL-6 [31,32,33]. In addition, proinflammatory cytokines, such as TNF-α and IL-6, are produced predominantly by monocytes and macrophages in the liver and are key factors in the development of NAFLD [34,35,36]. Furthermore, many studies have reported that the inhibition of TNF-α and IL-6 can decrease hepatic fat accumulation in NAFLD [37]. Our results showed that Ho reduced the infiltration of inflammatory cells (Figure 2A) as well as IL-6, IL-1β, and IL-6 gene expressions (Figure 3). These findings suggest that Ho has a protective effect on hepatic inflammation by reducing the proinflammatory cytokine levels produced in OVX mice.

Our results indicate that Ho ameliorated OVX-induced fatty liver and inflammation and the associated lipid metabolism in OVX-mice as an animal model for postmenopausal women. These findings suggest that Ho contributes to the amelioration of NAFLD in postmenopausal women.

## 4. Materials and Methods

### 4.1. Animals and Treatments

The C57BL/6 mice (12 weeks old, *n* = 40) were obtained from Charles River Korea (Seoul, Korea) and kept at the Korea Food Research Institute at a constant temperature (22 ± 2 °C) with a 12-h light/12-h dark cycle (7:00–19:00) and free access to water and food. Animal studies were carried out in accordance with institutional and national guidelines, and all animal experiments were performed according to standard guidelines by the Korea Food Research Institute Animal Care and Use Committee (IACUC#KFRI-M-17047). Thirty mice were ovariectomized (OVX), and 10 mice were subjected to incision and suturing without ovary removal (SHAM group). Two weeks after recovering from surgery, the OVX mice were divided into the following three groups: OVX mice treated with the vehicle (corn oil) (OVX group, *n* = 10), OVX rats treated with β-estradiol (Es; the OVX+Es group, *n* = 10), and OVX mice treated with Ho (the OVX+Ho group, *n* = 10). The experimental diets were based on the American Institute of Nutrition (AIN)-93M diets (Dyets, Bethlehem, PA, USA), and the OVX+Ho group was fed the AIN-93M diet with 50 mg Ho. The OVX+Es group was treated daily with Es (0.4 mg/kg) via oral gavage, and the other three groups were treated daily with the vehicle (corn oil). The treatments started 14 days after surgery and continued for an additional 84 days. Body weights were measured weekly. Mice were killed through cervical dislocation under anesthesia (10 mg/kg ketamine + 0.5 mg/kg xylazine), and whole blood was collected via cardiac puncture. After laparotomy, animals were culled, and liver and epidermal fats were weighed.

### 4.2. Serum Lipid Analysis

Serum TC, HDL-C, TC AST, and ALT were examined using commercial enzyme kits (Asan Pharmaceuticals, Hwasung, Korea). LDL-C was measured as follows: LDL-C = TC − HDL-C − TG/5.

### 4.3. Histological Analysis

Liver tissues were fixed in 4% formaldehyde and embedded in paraffin, and then sliced into 5 µm thick sections. Next, the liver tissues of each sample were stained using hematoxylin and eosin (H&E). Microscopy (100× magnification) with four randomly chosen non-overlapping fields was used to analyze the stained sections, which were assigned a liver inflammation score by an examiner using previously described methods. Slides were investigated and evaluated according to NAFLD activity scoring (NAS) [38]. Steatosis (0–3), lobular inflammation (0–2), and hepatocellular ballooning (0–2) were quantified, respectively.

### 4.4. Analysis of Gene Expression in Liver Samples by RT-PCR

Total RNA was isolated from the liver tissues using a Qiagen mini kit (Qiagen, Valencia, CA, USA). Quantitative real-time PCR was performed using the iTaq universal SYBR Green I supermix (Bio-Rad, Hercules, CA, USA), according to the supplier’s protocol. Data were normalized with GAPDH levels. The primer sequences are as follows: Interleukin (IL)-1β sense: 5′-TGT AAT GAA AGA CGG CAC ACC-3′, IL-1β antisense: 5′-TCT TCT TTG GGT ATT GCT TGG-3′; IL-6 sense: 5′-TGG AGT ACC ATA GCT ACC TGG A-3′. IL-6 antisense: 5′-TGA CTC CAG CTT ATC TGT TAG GAG-3′; Tumor necrosis factor (TNF)-α sense: 5′-ACC CTC ACA CTC AGA TCA TC-3′, TNF-α antisense: 5′-GAG TAG ACA AGG TAC AAC CC-3′; GAPDH sense, 5′-AAA TGG TGA AGC TCG CTC TG-3′, and GAPDH antisense, 5′-TGA AGG GGT CGT TGA TGG-3′.

### 4.5. Statistical Analysis

All data were analyzed using Prism (GraphPad Software, La Jolla, CA, USA). Values for animal experiments are presented as means ± standard error of mean. In vitro data are presented as means ± standard deviation. All statistical analyses were determined using *t*-tests. Statistical significance was considered when *p* < 0.05.

## Figures and Tables

**Table 1:** Serum blood parameter in OVX induced dyslipidemia. TC; total c, TG; triglycerides, HDL-C; high-density lipoprotein cholesterol, LDL-C; low-density lipoprotein. Values are given as means ± SEM.

	SHAM	OVX	OVX+Es	OVX+Ho
TC (mg/dL)	127.4 ± 22.1	197.7 ± 16.9 ^#^	191.5 ± 10.1	141.1 ± 14.1 *
HDL-C (mg/dL)	93.3 ± 21.6	75.6 ± 4.4	95.6 ± 8.9	75.8 ± 10.8
LDL-C (mg/dL)	23.1 ± 2.3	103.6 ± 13.4 ^##^	80.2 ± 7.7	65.2 ± 7.2 *
TG (mg/dL)	64.2 ± 6.3	88.0 ± 7.7	70.9 ± 6.0	56.22 ± 4.3 *

^#^ *p* < 0.05 and ^##^ *p* < 0.01 vs. control group. * *p* < 0.05 vs. OVX group.
